# Supplementary figures and images for: Current velocity, water quality, and benthic taxa as predictors for coral recruitment rates on the Great Barrier Reef
Source: PLoS One. 2025 Mar 26;20(3):e0319521. doi: 10.1371/journal.pone.0319521 (PMC11940690; doi:10.1371/journal.pone.0319521)

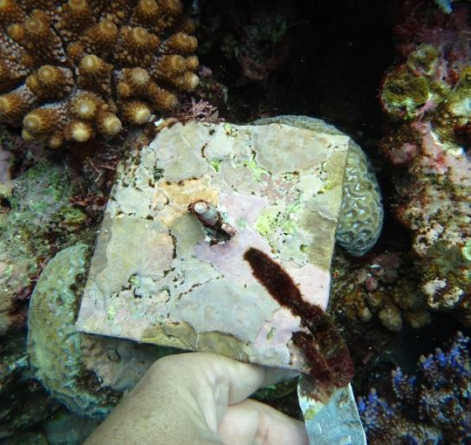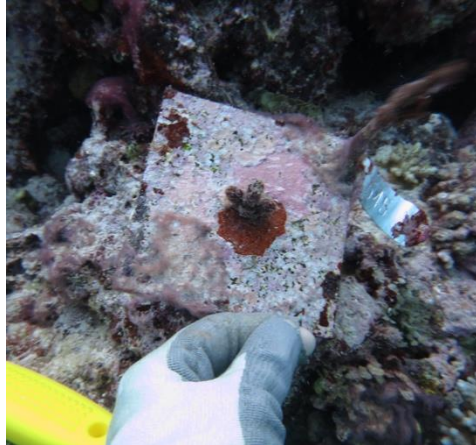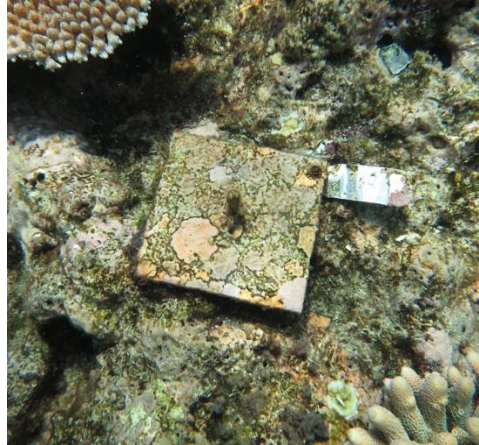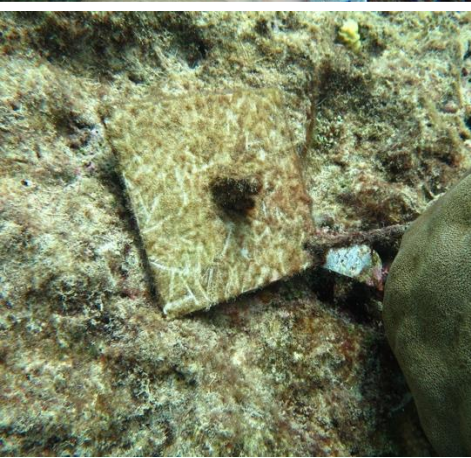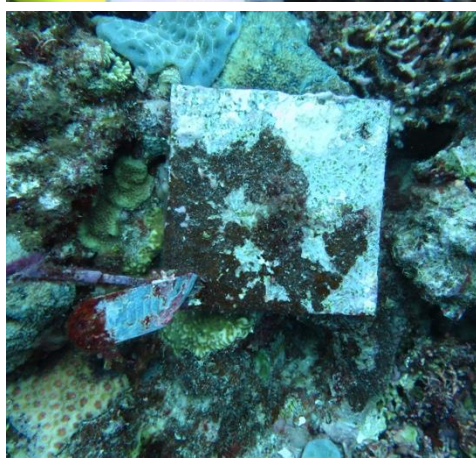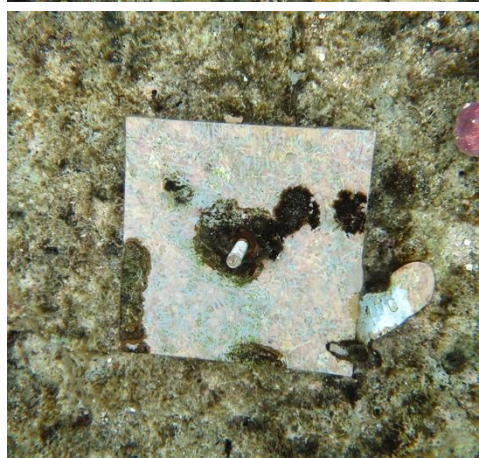

Supplement: S1 Fig — (PDF) [file pone.0319521.s001.pdf]

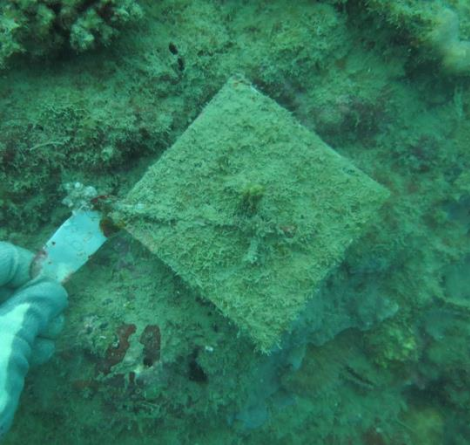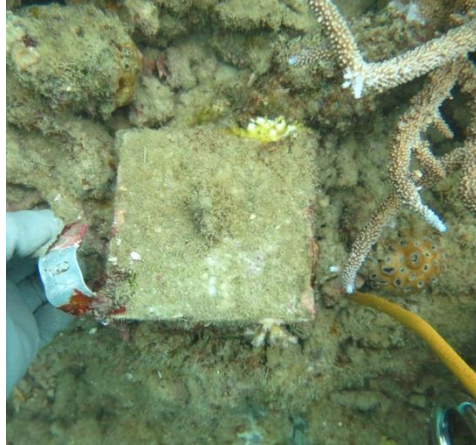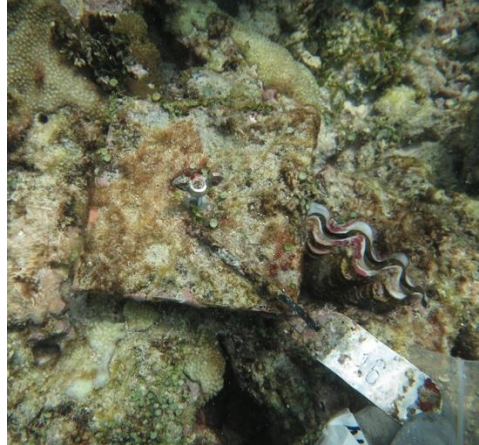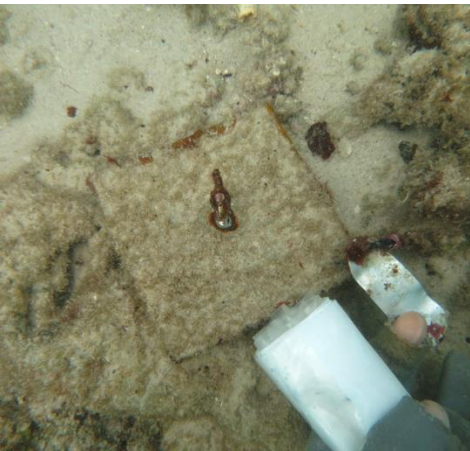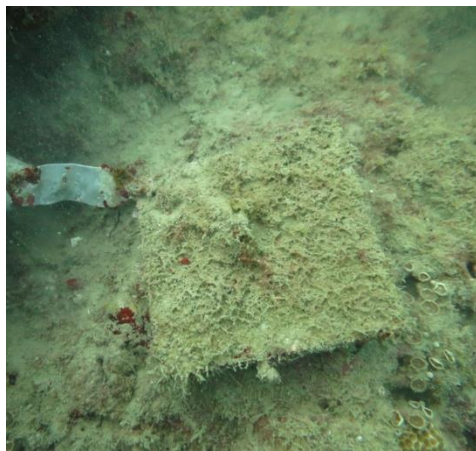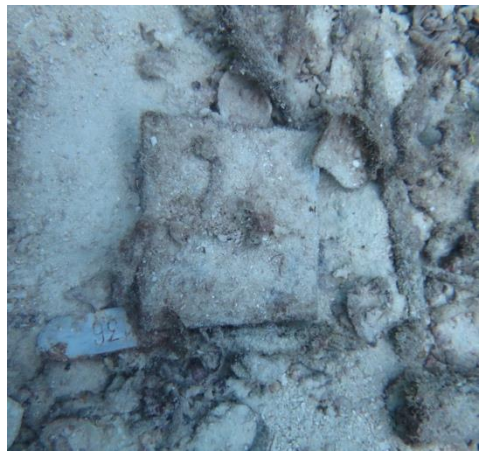

Supplement: S2 Fig — (PDF) [file pone.0319521.s002.pdf]

Prediction

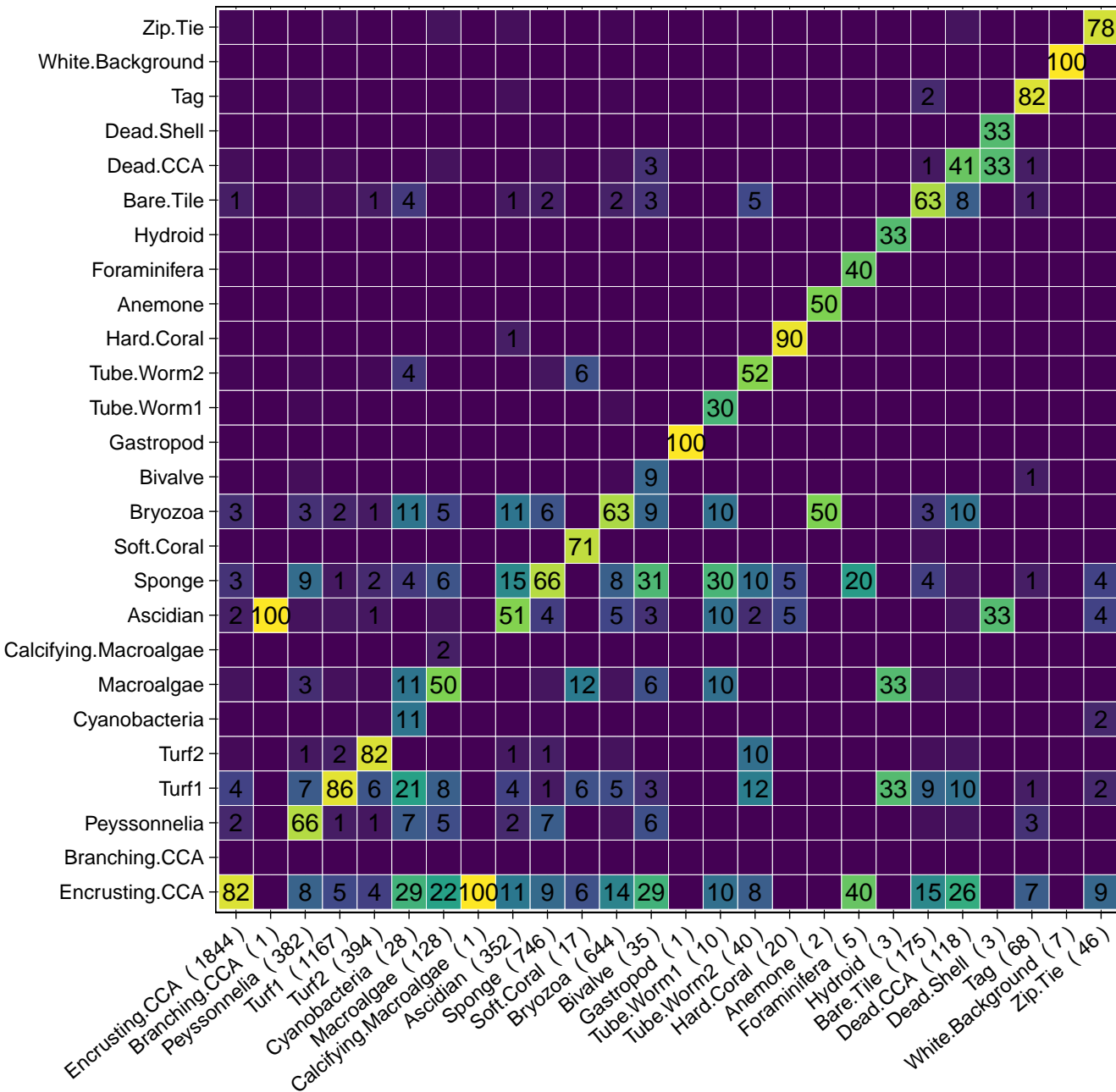

Actual (%)

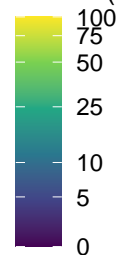

Actual

Supplement: S3 Fig — Human classified points were derived from a randomized subset of 25% of tiles. Numbers on the x-axis label indicate the number of points visually annotated per class. Values along the diagonal indicate the percentage of points accurately labeled by ReefCloud, values that vertically deviate from the diagonal line indicate a misclassification by ReefCloud. Cells with values ≥ 1% are labeled. (PDF) [file pone.0319521.s006.pdf]

Prediction

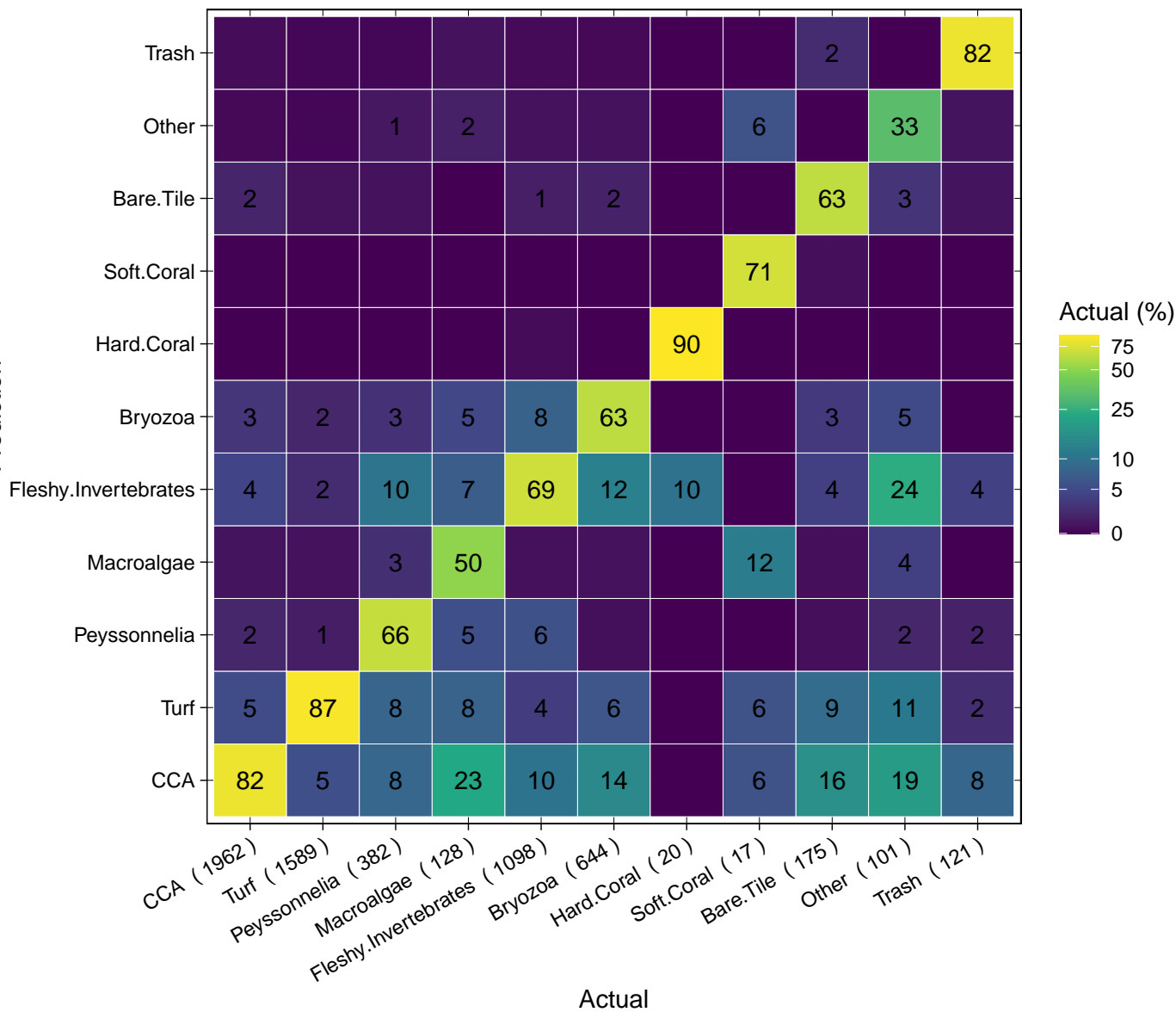

Supplement: S4 Fig — Mean weighted accuracy = 76.1%. (Legend as in S3 Fig). (PDF) [file pone.0319521.s007.pdf]
