# Supplementary material for: Current velocity, water quality, and benthic taxa as predictors for coral recruitment rates on the Great Barrier Reef
Source: PLoS One. 2025 Mar 26;20(3):e0319521. doi: 10.1371/journal.pone.0319521 (PMC11940690; doi:10.1371/journal.pone.0319521)
Supplement: S1 Table — Acronyms: FR = front reef, FL = flank reef, BA = back reef, LA = lagoon, D = deep (15m), S = shallow (5m), F = flat (1m). (DOCX) [file pone.0319521.s003.docx]

**S1 Table. Number of settlement tiles retrieved across the 7 regions, and 12 reefs, per site and depth.** Acronyms: FR = front reef, FL = flank reef, BA = back reef, LA = lagoon, D = deep (15m), S = shallow (5m), F = flat (1m).

| **Region** | **Reef** | **Deployment Time (months)** | **Site: Depth (in brackets: number of tiles)** | **Total number of tiles** |
| --- | --- | --- | --- | --- |
| Torres.Clear | Masig Island  (9°44'39.26"S, 143°24'56.75"E) | 23 | FR: D (10), S (9), F (5)  FL: S (4), F (4)  BA: D (10), S (10), F (9)  LA: S (5) | 118 |
|  | Aukane Reef  (9°52'3.41"S, 143°23'21.68"E) | 23 | FR: D (4), S (8), F (10)  FL: S (5), F (5)  BA: S (10), F (10) |  |
| Torres.Turbid | Dungeness Reef  (9°59'7.13"S, 142°54'30.74"E) | 23 | FR: S (10), F (5)  BA: S (12), F (15)  LA: S (5), F (2) | 49 |
| North.Clear | Lizard Island  (14°41'52.8"S, 145°26'43.8"E). | 20 | FR: S (10), F (10)  FL: S (5), F (2)  BA: S (10), F (9)  LA: S (5), F (5) | 133 |
|  | Moore Reef  (16°50'49.56"S, 146°13'2.64"E) | 24 | FR: D (9), S (7), F (7)  FL: D (5), S (5), F (2)  BA: D (10), S (9), F (8)  LA: D (5), S (5), F (5) |  |
| Central.Clear | Davies Reef  (18°49'52.50"S, 147°37'59.38"E) | 24 | FR: D (10), S (10), F (9)  FL: D (4), S (5), F (3)  BA: D (10), S (10), F (5)  LA: D S (8), F (5) | 131 |
|  | Chicken Reef  (18°39'37.31"S, 147°42'9.63"E) | 24 | FL: S (5), F (1)  BA: S (10), F (12) |  |
|  | Little Broadhurst Reef  (18°56'57.65"S, 147°41'34.54"E) | 24 | FL: S (5), F (4)  BA: S (5), F (5)  LA: S (5) |  |
| Central.Turbid | Palm Island Reefs  (18°33'2.88"S, 146°29'18.31"E) | 24 | FR: D (5), S (4), F (3)  FL: D (4), S (4), F (3)  BA: D (8), S (10), F (9) | 50 |
| South.Clear | Heron Island  (23°28'19.56"S, 151°58'40.8"E) | 24 | FR: D (10), S (10), F (8)  FL: S (4), F (1)  BA: D (10), S (9), F (6)  LA: S (5) | 101 |
|  | Lady Musgrave  (23°53'6.36"S, 152°24'49.32"E). | 24 | FL: S (10), F (8)  BA: S (8), F (7)  LA: S (5) |  |
| South.Turbid | Great Keppel Islands  (23°5'7.08"S, 150°53'56.76"E). | 24 | FL: S (3), F (4)  BA: S (19), F (14) | 40 |
