# Supplementary material for: Current velocity, water quality, and benthic taxa as predictors for coral recruitment rates on the Great Barrier Reef
Source: PLoS One. 2025 Mar 26;20(3):e0319521. doi: 10.1371/journal.pone.0319521 (PMC11940690; doi:10.1371/journal.pone.0319521)
Supplement: S2 Table — (DOCX) [file pone.0319521.s004.docx]

**S2 Table. Environmental and spatial predictors.**

| **Predictor** | **Units** | **Origin** | **Spatial Scale** |
| --- | --- | --- | --- |
| Total Nitrogen (TN) | mg N m^-3^ | eReefs biogeochemical model (version 3p1a) | 4x4 km^2^ |
| Total Alkalinity (TA) | mmol m^-3^ | eReefs biogeochemical model (version 3p1a) | 4x4 km^2^ |
| Aragonite Saturation State | mmol m^-3^ | eReefs biogeochemical model (version 3p1a) | 4x4 km^2^ |
| pH | Log(mM) | eReefs biogeochemical model (version 3p1a) | 4x4 km^2^ |
| Temperature | °C | eReefs hydrodynamic model (version H2p0 | 1x1 km^2^ |
| Salinity | psu | eReefs hydrodynamic model (version H2p0 | 1x1 km^2^ |
| Horizontal water velocity at the seabed (Ubed) | m s^-1^ | David Callaghan’s Model | 15x15 m^2^ |
| Secchi Depth | m | eReefs hydrodynamic model (version 3p1a) | 4x4 km^2^ |
| Sediment | mg cm^-2^ | *in situ* | 11.5x11.5 cm^2^ (per tile level) |
| Region | Torres Strait.Clear, Torres Strait.Turbid, North.Clear, Central.Clear, Central.Turbid, South.Clear, South.Turbid |  |  |
| Depth | Deep (D) = ~12m, Shallow (S) = ~5m,  Flat (F) = ~1m |  |  |
