# Supplementary material for: Current velocity, water quality, and benthic taxa as predictors for coral recruitment rates on the Great Barrier Reef
Source: PLoS One. 2025 Mar 26;20(3):e0319521. doi: 10.1371/journal.pone.0319521 (PMC11940690; doi:10.1371/journal.pone.0319521)
Supplement: S3 Table — (DOCX) [file pone.0319521.s005.docx]

**S3 Table. Label set categories used in ReefCloud to annotate the settlement tile communities, and their categorization into benthic functional groups used in the study.**

| **Label Set Categories** | **Benthic Functional Groups** |
| --- | --- |
| Encrusting.CCA | Crustose coralline algae (CCA) |
| Branching.CCA* | Branching.CCA* |
| Dead.CCA | Crustose coralline algae (CCA) |
| *Peyssonnelia* | *Peyssonnelia* spp. |
| Turf1 | Turf |
| Turf2 | Turf |
| Cyanobacteria | Turf |
| Macroalgae (fleshy)* | Macroalgae* |
| Calcifying.Macroalgae* | Calcifying.Macroalgae* |
| Ascidian | Fleshy Invertebrate |
| Sponge | Fleshy Invertebrate |
| Soft.Coral | Soft.Coral |
| Bryozoa | Bryozoa |
| Bare.Tile | Bare.Tile |
| Anemone* | Anemone* |
| Bivalve* | Bivalve* |
| Gastropod* | Gastropod* |
| Tube.Worm1* | Tube.Worm1* |
| Tube.Worm2* | Tube.Worm2* |
| Hard.Coral* | Hard.Coral* |
| Foraminifera* | Foraminifera* |
| Egg* | Egg* |
| Hydroid^*^ | Hydroid^*^ |
| Dead.Shell* | Dead.Shell* |
| Tag* | Trash* |
| Tile.Center* | Trash* |
| Black.Background* | Trash* |
| White.Background* | Trash* |
| Ziptie* | Trash* |

^*^ label set categories not included in analysis
