# Supplementary material for: Current velocity, water quality, and benthic taxa as predictors for coral recruitment rates on the Great Barrier Reef
Source: PLoS One. 2025 Mar 26;20(3):e0319521. doi: 10.1371/journal.pone.0319521 (PMC11940690; doi:10.1371/journal.pone.0319521)
Supplement: Attachment — Submitted filename: Response to reviewers.docx [file pone.0319521.s009.docx]

Response to Reviewers

Dear editors Tzen-Yuh Chiang and Ronalyn M. Ramos and anonymous reviewer,

Thank you for taking the time to review our manuscript “Current velocity, water quality, and benthic taxa as predictors for coral recruitment.” We are grateful to have received your constructive comments, which have helped us improve our manuscript. We have thoroughly reviewed our manuscript and taken into consideration each of your comments. Below, we have responded to each comment individually with our response in italics and examples of changes to the text in blue.

In addition to changes requested by the editor and reviewer, we have also made minor changes to further improve clarity in the manuscript. We have changed the title from “Current velocity, water quality, and benthic taxa as predictors for coral recruitment” to “Current velocity, water quality, and benthic taxa as predictors for coral recruitment rates on the Great Barrier Reef”, better placing our study. Minor changes in word choice have been made throughout the text for greater clarity, for example we changed the name of one of our benthic predictors from “invertebrates” to “fleshy invertebrates” to emphasize the separation between this functional group (ascidians and sponges) to that of bryozoans. In addition, we have updated Table 2 recruit densities from global (global regions including GBR) to global (all other regions, excluding the GBR) to emphasize the differences in recruit density between the GBR and other global regions.

Once again, thank you for your valuable input. We look forward to your comments on the revised version of our submission.

**With regards to PLOS ONE journal requirements:**

1. Please ensure that your manuscript meets PLOS ONE's style requirements, including those for file naming.

***Response****: We have thoroughly reviewed PLOS ONE’s style requirements and have made minor changes to adhere to these standards. All instances of “Figure” have been changed to “Fig”. Supporting information captions have been moved below the References section. Text in Fig1 was not an approved font and has promptly been changed to the approved Arial font. We believe that the manuscript, including file names, now follows all PLOS ONE’s style requirements.*

2. We note that the grant information you provided in the ‘Funding Information’ and ‘Financial Disclosure’ sections do not match.

***Response:*** *The Financial Disclosure is correct and the Funding Information section of the application will be edited upon revised manuscript submission.*

3. We note that you have provided funding information [in the Acknowledgments Section] that is not currently declared in your Funding Statement. However, funding information should not appear in the Acknowledgments section or other areas of your manuscript. We will only publish funding information present in the Funding Statement section of the online submission form.

Please remove any funding-related text from the manuscript and let us know how you would like to update your Funding Statement.

***Response:*** *The funding information we have provided in the Funding Statement is correct, and we have now deleted the funding statement from the Acknowledgements: “We acknowledge the Aboriginal and Torres Strait Islander Traditional Owners of the sea Country where this work was conducted and are grateful for their consent to conduct this research. We thank Christopher Doropoulos and Renata Ferrari for their invaluable contributions in reviewing an earlier version of this manuscript, and to Mathilda Bates for her assistance in data collection.”*

4. When completing the data availability statement of the submission form, you indicated that you will make your data available on acceptance. We strongly recommend all authors decide on a data sharing plan before acceptance, as the process can be lengthy and hold up publication timelines. Please note that, though access restrictions are acceptable now, your entire data will need to be made freely accessible if your manuscript is accepted for publication. This policy applies to all data except where public deposition would breach compliance with the protocol approved by your research ethics board. If you are unable to adhere to our open data policy, please kindly revise your statement to explain your reasoning and we will seek the editor's input on an exemption. Please be assured that, once you have provided your new statement, the assessment of your exemption will not hold up the peer review process.

***Response:*** *Yes, we understand that allowing access to data after acceptance may hold up publication timelines. We will be allowing public availability of our data sets which are prepared and ready to be published for a speedy upload upon acceptance.*

5. Please include your full ethics statement in the ‘Methods’ section of your manuscript file. In your statement, please include the full name of the IRB or ethics committee who approved or waived your study, as well as whether or not you obtained informed written or verbal consent. If consent was waived for your study, please include this information in your statement as well.

***Response:*** *We have added “Ethics statement” to the “Methods” section of the manuscript. This section adds the permit issued by the Great Barrier Reef Marine Park Authority (G21/44774.1) to conduct our field research and sample collections. We have highlighted that our study did not require an animal ethics committee approval as targeted and sampled organisms were non-cephalopod and non-crustacean invertebrates and are not under the definition of “animal” (i.e. non-human vertebrate or cephalopod).*

6. We note that Figure 1 in your submission contain [map/satellite] images which may be copyrighted. All PLOS content is published under the Creative Commons Attribution License (CC BY 4.0), which means that the manuscript, images, and Supporting Information files will be freely available online, and any third party is permitted to access, download, copy, distribute, and use these materials in any way, even commercially, with proper attribution. For these reasons, we cannot publish previously copyrighted maps or satellite images created using proprietary data, such as Google software (Google Maps, Street View, and Earth). AND Please clarify which map types you have used as https://s2maps.eu/# lists some as CC-BY SA or CC-BY NC which is not compliant with our licensing rules.

***Response:*** *We apologize for the potential misunderstanding of the licensure availability of our sources in Fig 1. Data sources used from EOX IT Services GmbH (Sentinel-2 Cloudless 2016) and AIMS, per these sources EOX IT (https://s2maps.eu), AIMS Sentinel 2 Composite (*[*https://eatlas.org.au/geonetwork/srv/eng/catalog.search#/metadata/c38d2227-25c0-4d1e-adbc-bddb4aac1929/formatters/xsl-view?root=div&view=advanced*](https://eatlas.org.au/geonetwork/srv/eng/catalog.search#/metadata/c38d2227-25c0-4d1e-adbc-bddb4aac1929/formatters/xsl-view?root=div&view=advanced)*), are CC BY 4.0 and are the only sources used in the updated Figure 1. Fig 1 has been simplified with the removal of unmodified 2024 Copernicus Sentinel-2 data. Fig 1 caption has been reworded to emphasize that all data used are under CC BY 4.0 license and clarifies which EOX IT cloudless layer was used (2016).*

7. Please review your reference list to ensure that it is complete and correct. If you have cited papers that have been retracted, please include the rationale for doing so in the manuscript text, or remove these references and replace them with relevant current references. Any changes to the reference list should be mentioned in the rebuttal letter that accompanies your revised manuscript. If you need to cite a retracted article, indicate the article’s retracted status in the References list and also include a citation and full reference for the retraction notice.

***Response:*** *We do not need to cite retracted articles but have added several new references in response to the reviewer and when doing a final reference completion check.*

**With regards to the anonymous reviewers’ main feedback:**

1. On the use of the settlement plates, the authors claimed that the use of PVC materials can result in settlement pattern more similar to natural assemblages. However, Nozawa et al. (2011) found that plates with crevices can also enhance coral recruits. Although this study has not used this kind of coral recruit settlement plate, I would suggest the author to cite this reference that plates with crevice microstructures can enhance coral recruitment.

   Nozawa et al 2011 - Reconsideration of the Surface Structure of Settlement Plates Used in Coral Recruitment Studies – Zoological Studies 50(1): 53-60

***Response****: Micro-crevices assist with mimicking natural rugosity in short term tile deployment studies where benthic communities are not well developed on the tiles, helping to enhance coral recruitment. But recent research (dela Cruz and Harrison, 2024; doi:10.1016/j.jembe.2024.152029) has shown that over time, "flat" surfaces like PVC get bio fouled which creates rugosity and makes differences among different tile materials in long term studies such as ours negligible.*

2. Any morphological features to aid identification of coral recruits? Have this features used in training the AI? I would suggest you can cite the reference for recruit identifications. An example is:

   Babcock et al. 2003 - Identification of Scleractinian Coral Recruits from Indo-Pacific Reefs. Zoological Studies 42(1): 211-226

***Response:*** *Babcock et al. 2003 is indeed the source our coral ID observers used to aid in morphological identification of the coral recruits. We have added this reference to the methods section. The AI was not able to perform at the genera level of taxonomic resolution, although it could identify “large” areas of recruits as hard or soft coral. This was due to the fact that many recruits were on the millimeter scale (undetectable in our images) and required microscopy to be identified.*

3. The authors pointed out effect of environmental factors on coral recruitment received scant attentions. I would suggest in addition to the statistical analysis conducted in the present study, to use multivariate analysis to generate a separated nMDS patterns for the environmental and biological assemblages. This can be use the multivariate software PRIMER. Then use the Bio-Env analysis to match the environmental and biological assemblages to see any significant correlation and deduce the best subset of env. Factors which are more responsible for the biological assemblages. This result can further strengthen the PCA plots which the author conducted in the current manuscript. The strong side of this analysis can reflect whether the environment and biological assemblages are different among the sites and then correlate the two patterns together

***Response:*** *We appreciate your recommendation to use separate nMDS analyses and the Bio-Env approach to explore the relationship between environmental and biological assemblages.*

*Here, we have used boosted regression trees (BRT), which automatically select the most important environmental predictors based on their ability to explain variation in biological data. This method is particularly advantageous as it can account for complex, non-linear relationships and interactions between environmental variables and biological assemblages, making it a more robust approach for identifying key environmental drivers compared to traditional nMDS analysis.*

*Additionally, we employed Redundancy Analysis (RDA), which visually represents the relationship between environmental factors and biological assemblages in a 2D space. RDA provides a clear summary of how environmental variables influence biological patterns. To further substantiate these findings, we conducted PERMANOVA to test the statistical significance of the relationship between the environmental and biological data, thus confirming the strength of the patterns observed.*

*We believe that the combination of BRT, RDA, and PERMANOVA sufficiently addresses the reviewer's request by identifying important environmental predictors and demonstrating their relationship with biological assemblages. These methods allow us to provide a comprehensive understanding of the environmental influences on coral recruitment, without the need for separate nMDS or Bio-Env analyses.*

4. Effect of light on coral recruitments, there are examples in the Pacific that light can affect recruitment as the tropical and subtropical latitudinal waters differs in light intensity. The authors can consider reading and citing the following relevant references in their discussion for comparisons.

   Maida MJ, Coll C, Sammarco PW: Shedding light on scleractinian coral recruitment. J Exp Mar Biol Ecol 1994, 180: 189–202. 10.1016/0022-0981(94)90066-3

   Maida M, Sammarco PW, Coll JC: Effects of soft corals on scleractinian coral recruitment. I: directional allelopathy and inhibition of settlement. Mar Ecol Prog Ser 1995, 121: 191–202.

   Ho, MJ., Dai, CF. Coral recruitment of a subtropical coral community at Yenliao Bay, northern Taiwan. Zool. Stud. 53, 5 (2014). <https://doi.org/10.1186/1810-522X-53-5>
   ***Response:*** *Thank you for this suggestion, two of these references have now been added to the manuscript.*
5. What is the unit in the Y axis of figure 4?

***Response:*** *The y-axis of Fig 4 reflects the total recruit abundance or richness (#) per tile. The y-axis has been changed to “Recruit abundance and richness (tile^-1^)” for clarity.*

**With regards to the reviewers’ attached comments (by line of the first submission):**

1. Line 41: Include their ecological significance and cultural.

***Response****: Done, we have now included more information on coral reefs ecological and cultural significance as follows: “Coral reefs are the Earth’s most diverse marine ecosystems, serving as a critical habitat and nursery to thousands of species while providing socioeconomic benefits to millions of people through for instance, cultural heritage, fisheries, tourism, protection against coastal erosion [1]”*

2. Line 43: Explain what types of disturbances.

***Response****: Thank you for this suggestion, we have now added more specifics of types of disturbances affecting coral reefs as follows: “…disturbances, such as cyclones, marine heat waves, mass bleaching, disease, and crown of thorns outbreaks, quashing coral populations”*

3. Line 49: I think the intro paragraph needs to be more captivating

***Response:*** *We have now substantially rewritten the Introduction section, and believe it is now more captivating and outlining a better rationale for our study.*

4. Line 55: This paragraph is quite short. Either explain more with details or include it with another paragraph

*Response: Yes, we agree. We have now added more details to this paragraph on hydrodynamics (in the Introduction section).*

5. Line 58: Sedimentation. Where is this sediment coming from? Erosion?

***Response:*** *While sources of sediment runoff can be tricky to pinpoint, research points towards erosion from land use change (particularly towards grazelands) to be the greatest contributor of increased of sediment loads. This information has now been added to the text. Changed word choice of sediments to sedimentation.*

6. Line 59: Reefs closer to coastal zones may be more affected by sedimentation and nutrient run-off. Include a reference.

**Response**: Agreed. This section of the introduction was altered and more details and references on the origins of sediment which may more greatly affect coastal zones were added: *“Off the Northeast Australian coast, the degradation of water quality, especially on reefs near the coast, soil erosion from beef grazing lands and fertilizer application to sugarcane cultivation [24-26].”*

7. Line 60: Reference?

***Response:*** *We have added a reference to Fabricius, 2005 (doi: 10.1016/j.marpolbul.2004.11.028).*

8. Line 69: Put this paragraph after first paragraph.

***Response****: Done.*

9. Line 85: example of genera (e.g., Neogoniolithon)

***Response****: Done.*

10. Line 157: Why did you put them out haphazardly? Cite what the research benefit may be and why you choose that method.

***Response****: “Haphazardly” was an incorrect word choice, thank you for catching this. Tiles had to be fixed onto hard non-living consolidated reef substratum, which was sometimes difficult to find. Therefore, tiles were placed within the range of 1 to 5 meters from one another due to this constraint. The amended sentence reads: “At each site and depth level, five replicate tiles were deployed approximately 1-5 m distance between each other in a cluster and fixed on firm substratum.”*

11. Line 165: the benthic taxa could have changed throughout time. Were the photos takes of the tiles in between the timeframe for comparison?

***Response****: We agree that monitoring changes of the tiles through time would have been interesting, however this was not possible due to the sheer size of the research conducted, with 141 stations across >1000 km, and was beyond the scope of this study. For our data, we were able to obtain the one time point after approximately two years of deployment at sea.*

12. Line 165: what happened to the other 88 tiles? It's really important to explain what why 88 tiles weren't used in the data analysis, whether they were lost or something else.

***Response****: Yes, the 88 other tiles came loose from the substratum and were lost. This information is now added to the text: “...622 of the original tiles were retrieved while 88 were lost due to dislodgement.”*

13. Line 173: How were they identified and who? Coral taxonomist experts?

***Response****: The coral identifications were made using a microscope within hours after the tile retrieval using Babcock 2003 as a guide. They were conducted by individuals with years of coral identification experience, who have conducted multiple JCU workshops and seminars in coral ID. The text was amended to include who identified the coral recruits as follows: “Coral taxonomic identification, made by observers experienced with coral identification, using morphological features of the corallites, was recorded where possible, limited to genus and family level or unknown [55].”*

14. Line 177: Write out the light setting. The methods should be clear enough so future researchers can replicate your methods and cite you.

***Response****: Thank you for making this point on reproducibility of the methods. We have provided additional information in the methods on the number, make and model of the strobes that we used in the study: “Pictures were taken using a Nikon D300 camera and 60 mm NIKKOR lens linked with a set of two remote controlled Nikon SB-R200 strobes.”*

15. Line 234: It would be good to explain how the data for this model was collected. What is the time frame being analyzed for it to be considered long-term? AND Line 244: This is a good. Put these dates at the beginning if this section.

***Response****: Dates were moved to the beginning of the section. The origin of the eReefs models is described in the methods as built from wind, rainfall, pressure, air, and dewpoint temperature, collected from the Bureau of Meteorology.*

16. Line 302: Within the experiment or at the end? The methods section made it appear as if the tiles were collected and observed only at the end of the study.

***Response****: Yes at the end, text amended for clarity: “After approximately two years of deployment…”*

17. Line 304: CCA are diverse and specific subfamilies and genera are known to be preferred for coral recruitment/settlement. (e.g., Gomez-Lemos et al. 2018; Deinhart et al. 2022; Abdul Wahab et al. 2023) AND Line 418: If you still have these tiles, it would be good to identify what CCA species they are (DNA and/or morphological). CCA species will tell you a lot more as to why there aren't coral recruits associated to that substrate.

***Response:*** *Yes, while we agree and appreciate the different roles of CCA species, this research was beyond the scope of the present study. Identification would require ontogenetics or microscopic identification and due to the sheer number of samples (622 tiles), and available resources, these methods were not feasible. In addition, our AI is not presently capable of identification at this fine of a scale. We have added a sentence in the discussion to reflect this.*

18. Line 332: Were there differences in coral genera?

***Response****: The primary coral genera in all seven regions were Acropora, following Pocillopora, and Porites, except in the South.Turbid where there were no Porites.* These details have been added to the text.

19. Line 412: How is this often assumed to be recruitment substrate? CCA are well known to be preferred recruitment substrate.

***Response****: The assumption that bare tile is often presumed to be a preferred settlement substrate was removed from the results. We have further defined “bare tile” to inform the reader that this substrate type is often overgrown by biofilms but free of macrobenthos.*

20. Line 440: If you compare to global, you need to cite it.

***Response:*** *Yes, we agree. This data is cited in Table 2 and text is adjusted for clarity that both global and GBR specific data are located there.*

21. Line 609: If you are aiming to write a whole section about it, it needs to be expanded and have more detail, otherwise include it with the rest of the discussion.

***Response****: Thank you, we have removed both the AI and Conclusion section titles.*

22. All your figures need a better resolution.

***Response:*** *All figures adhere to PLOS ONE’s guidelines, including those for resolution. When downloading the manuscript, a low resolution file is provided with the figures, a high resolution download can be made if you click the access/download button in the top right corner of the figures section.*

23. Fig 2: It would be good to have the measurements (ruler) in the photos of the tiles

***Response:*** *Yes, we have updated Fig 2 with digital scale bars.*

24. Fig 3: These color are too similar and not accessible to all readers. This is a good website to test your color palettes. <https://davidmathlogic.com/colorblind/#%23B3B3EC-%23FFC20A-%230C7BDC-%23AD5463-%23669877-%23CC6677-%23AA4499-%23882255>.

***Response:*** *Fig 3 has been updated with the “Tol_muted” 10 color accessible palette; color codes as outlined in the link provided by the reviewer.*

25. Fig 4: Is there supposed to be a plot here (blank space)?

***Response****: No, this blank space reflects that there was no turbid region in the northern GBR in our study, we wanted each region to be plotted next to its clear or turbid water pair. We have moved the x-axis up to make it clearer that there is not supposed to be a plot in this area.*
